# Impact of age on the management and prognosis of esophageal fistula after atrial fibrillation ablation—a subanalysis of the worldwide POTTER-AF study

**DOI:** 10.3389/fcvm.2025.1708499

**Published:** 2026-02-11

**Authors:** Sorin S. Popescu, Zeynep G. Demirtakan, Vanessa Schmidt, Helmut Pürerfellner, Philipp Sommer, Christian Sohns, Christian Veltmann, Daniel Steven, K. R. Julian Chun, Philippe Maury, Estelle Gandjbakhch, Mikael Laredo, Stephan Willems, Thomas Beiert, Leon Iden, Anna Füting, Raphael Spittler, Sergio Richter, Anja Schade, Malte Kuniss, Carsten Wunderlich, Dong-In Shin, Dirk Grosse Meininghaus, Marc Bonsels, David Reek, Uwe Wiegand, Alexander Bauer, Andreas Metzner, Lars Eckardt, Olaf Krahnefeld, Christian Sticherling, Michael Kühne, Dinh Quang Nguyen, Laurent Roten, Dominik Linz, Pepijn van der Voort, Bart A. Mulder, Johan Vijgen, Alexandre Almorad, Charles Guenancia, Laurent Fauchier, Serge Boveda, Yves De Greef, Antoine Da Costa, Pierre Jais, Antoine Milhem, Laurence Jesel, Rodrigue Garcia, Hervé Poty, Ziad Khoueiry, Julien Seitz, Julien Laborderie, Alexis Mechulan, Francois Brigadeau, Alexandre Zhao, Yannick Saludas, Olivier Piot, Nikhil Ahluwalia, Claire Martin, Jian Chen, Bor Antolic, Georgios Leventopoulos, Emin Evren Özcan, Hikmet Yorgun, Serkan Cay, Kivanc Yalin, Maichel Sobhy Botros, Ewa Jędrzejczyk-Patej, Osamu Inaba, Ken Okumura, Koichiro Ejima, Houman Khakpour, John N. Catanzaro, Vivek Reddy, Andrea Natale, Hermann Blessberger, Bing Yang, Julia Vogler, Karl-Heinz Kuck, José Luis Merino, Ahmad Keelani, Christian-H. Heeger, Roland Richard Tilz

**Affiliations:** 1 Department of Rhythmology, University Heart Center Lübeck, University Hospital Schleswig-Holstein, Lübeck, Germany; 2 German Center for Cardiovascular Research (DZHK), Partner Site Hamburg/Kiel/Lübeck, Lübeck, Germany; 3 Institute for Diagnostic and Interventional Radiology, Faculty of Medicine and University Hospital Cologne, University of Cologne, Cologne, Germany; 4 Department of Electrophysiology, Ordensklinikum Linz Elisabethinen, Linz, Austria; 5 Kliniken für Elektrophysiologie/Rhythmologie, Herz- und Diabeteszentrum NRW, Universitätsklinik der Ruhr-Universität Bochum, Bad Oeynhausen, Germany; 6 Heart Center Bremen, Electrophysiology Bremen, Bremen, Germany; 7 Department for Electrophysiology, Heart Center University Cologne, Cologne, Germany; 8 MVZ CCB am Agaplesion Markus Krankenhaus, Frankfurt a.M., Germany; 9 Department of Cardiology, University Hospital Rangueil, Toulouse, France; 10 Sorbonne Université, APHP, Pitié Salpêtrière University Hospital, Cardiology Institute, Paris, France; 11 Klinik für Kardiologie und Internistische Intensivmedizin, Asklepios Klinik St. Georg, Hamburg, Germany; 12 Heart Center Bonn, Department of Internal Medicine II, University Hospital Bonn, Bonn, Germany; 13 Heart Center, Segeberger Kliniken (Academic Teaching Hospital of the Universities of Kiel, Lübeck and Hamburg), Bad Segeberg, Schleswig-Holstein, Germany; 14 Department of Electrophysiology, Alfred Krupp Hospital, Essen, Germany; 15 Department of Medicine, Witten/Herdecke University, Witten, Germany; 16 Department of Cardiology II/Electrophysiology, Center for Cardiology, University Hospital Mainz, Mainz, Germany; 17 Department of Internal and Cardiovascular Medicine, Herzzentrum Dresden, University Clinic, Technische Universität Dresden, Dresden, Germany; 18 Department of Interventional Electrophysiology, Helios Hospital Erfurt, Erfurt, Germany; 19 Department of Rhythmology, Rhoen Klinikum Campus Bad Neustadt/Saale, Bad Neustadt/Saale, Germany; 20 Department of Cardiology, Kerckhoff Heart Center, Bad Nauheim, Germany; 21 Helios Klinikum Pirna, Klinik für Innere Medizin II, Pirna, Germany; 22 Department of Cardiology, Heart Centre Niederrhein, Helios Clinic Krefeld, Krefeld, Germany; 23 Department of Cardiology, Medical University Lausitz—Carl Thiem, Cottbus, Germany; 24 Kliniken Maria Hilf GmbH, Mönchengladbach, Germany; 25 Department of Cardiology, University Hospital Augsburg, Augsburg, Germany; 26 Sana-Klinikum Remscheid GmbH, Akademisches Lehrkrankenhaus der Universität zu Köln, Remscheid, Germany; 27 Diak-Klinikum Schwäbisch Hall und Klinikum Crailsheim, Schwäbisch Hall, Germany; 28 Universitäres Herz- und Gefäßzentrum, Klinik für Kardiologie, Universitätsklinikum Hamburg-Eppendorf, Hamburg, Germany; 29 Department of Cardiology II (Electrophysiology), University Hospital Münster, Münster, Germany; 30 Sana Kliniken Lübeck, Lübeck, Germany; 31 Department of Cardiology, University Hospital Basel, Basel, Switzerland; 32 St. Vinzenz-Hospital Köln, Köln, Germany; 33 Department of Cardiology, Inselspital, Bern University Hospital, University of Bern, Bern, Switzerland; 34 Department of Cardiology, Maastricht University Medical Center and Cardiovascular Research Institute Maastricht, Maastricht, Netherlands; 35 Catharina Hospital, Eindhoven, Netherlands; 36 Department of Cardiology, University Medical Center Groningen, University of Groningen, Groningen, Netherlands; 37 Heart Center Hasselt, Jessa Hospital, Hasselt, Belgium; 38 Heart Rhythm Management Centre, Postgraduate Program in Cardiac Electrophysiology and Pacing, Universitair Ziekenhuis Brussel—Vrije Universiteit Brussel, European Reference Networks Guard-Heart, Brussels, Belgium; 39 Cardiology Department, Dijon University Hospital, Dijon, France; 40 Service de Cardiologie, Centre Hospitalier Universitaire Trousseau, Tours, France; 41 Cardiology—Heart Rhythm Management Department, Clinique Pasteur, Toulouse, France; 42 Department of Cardiology, ZNA Heart Centre, Antwerp, Belgium; 43 Division of Cardiology, Jean Monnet University, Saint-Etienne, France; 44 CHU Bordeaux, University of Bordeaux, Bordeaux, France; 45 La Rochelle Hospital, La Rochelle, France; 46 University Hospital Strasbourg, Strasbourg, France; 47 Department of Cardiology, University Hospital of Poitiers, Poitiers, France; 48 Centre d’Investigation Clinique 1402, University Hospital of Poitiers, Poitiers, France; 49 Clinique Tonkin, Lyon, France; 50 Clinique Saint Pierre, Perpignan, France; 51 Hospital St. Joseph, Marseille, France; 52 Bayonne Hospital, France; 53 Hospital Clairval, Marseille, France; 54 University Hospital Lille, France; 55 Clinique Ambroise Parée, Paris, France; 56 Clinique Pôle Santé République, Clermont Ferrand, France; 57 Centre Cardiologie du Nord, Saint Denis, France; 58 Barts Heart Centre, Barts Health NHS Trust, London, United Kingdom; 59 William Harvey Heart Centre, Queen Mary University of London, United Kingdom; 60 Royal Papworth Hospital, Cambridge, United Kingdom; 61 Department of Heart Disease, Haukeland University Hospital, University of Bergen, Bergen, Norway; 62 Department of Cardiology, University Medical Center Ljubljana, Ljubljana, Slovenia; 63 University of Patras, Greece; 64 Heart Rhythm Management Center, Dokuz Eylul University, Izmir, Türkiye; 65 Department of Cardiology, Hacettepe University, Ankara, Türkiye; 66 Department of Cardiology, Division of Arrhythmia and Electrophysiology, University of Health Sciences, Yuksek Ihtisas Cardiovascular Building, Ankara City Hospital, Ankara, Türkiye; 67 Cerrahpasa Faculty of Medicine, Department of Cardiology, Istanbul University-Cerrahpasa, Istanbul, Türkiye; 68 Department of Critical Care Medicine, Faculty of Medicine, Cairo University, Cairo, Egypt; 69 Department of Cardiology, Congenital Heart Diseases and Electrotherapy, Silesian Centre for Heart Diseases, Zabrze, Poland; 70 Department of Cardiology, Japanese Red Cross Saitama Hospital, Japan; 71 Division of Cardiology, Saiseikai Kumamoto Hospital, Kumamoto, Japan; 72 Department of Cardiology, Tokyo Women’s Medical University, Shinjuku-ku, Tokyo, Japan; 73 UCLA Cardiac Arrhythmia Center, Los Angeles, CA, United States; 74 Donald and Barbara Zucker School of Medicine at Hofstra/Northwell, Hempstead, NY, United States; 75 Department of Cardiology, Mather Hospital, Port Jefferson, NY, United States; 76 Helmsley Electrophysiology Center, Mount Sinai Fuster Heart Hospital, Icahn School of Medicine at Mount Sinai, New York, NY, United States; 77 St. David’s Medical Center, Texas Cardiac Arrhythmia Institute, Austin, TX, United States; 78 Department of Biomedicine and Prevention, Division of Cardiology, University of Tor Vergata, Rome, Italy; 79 Metro Health Medical Center, Case Western Reserve University School of Medicine, Cleveland, OH, United States; 80 Department of Cardiology, Kepler University Hospital, Linz, Austria; 81 Department of Cardiology, Shanghai East Hospital, Tongji University, Shanghai, China; 82 La Paz University Hospital, Universidad Autónoma de Madrid, Idipaz, Madrid, Spain

**Keywords:** age, atrial fibrillation, catheter ablation, complication, esophageal fistula

## Abstract

**Background:**

Esophageal fistula (EF) is a rare but devastating complication following atrial fibrillation (AF) ablation. Data regarding the impact of age on EF are scarce.

**Objective:**

To study the impact of age on the management and prognosis of EF following catheter ablation for AF.

**Methods:**

The POTTER-AF study is a worldwide registry on EF following catheter ablation for AF. A total of 553,729 patients underwent AF ablation in 214 centers between 1996 and 2022. Of them, 138 patients experienced EF, and data regarding age, management, and prognosis were available in 113 patients. The population was divided based on the median age.

**Results:**

The median age was 63 years; 54 patients were <63 years old (Group 1), and 59 patients were ≥63 years old (Group 2). The groups were similar regarding procedural characteristics. The older population had a shorter time to symptom onset [15.0 (6.0, 21.0) vs. 21.0 (10.0, 25.3) days; *p* = 0.031]. Group 2 was less likely to receive a brain CT or MRI for diagnosis (25.9% vs. 45.3%; *p* = 0.046). The older population was more likely to undergo endoscopic treatment without surgery (27.6% vs. 11.3%; *p* = 0.035). Conservative and surgical treatments were used in similar proportions. A trend toward higher fatality was noted in the older patients (72.9% vs. 56.6%; *p* = 0.078).

**Conclusion:**

The older population had a shorter time to symptom onset, was less likely to receive a brain CT or MRI, and more likely to be treated by an endoscopic approach only. The older patient group showed a trend toward a higher fatality.

## Graphical Abstract

**Figure:**
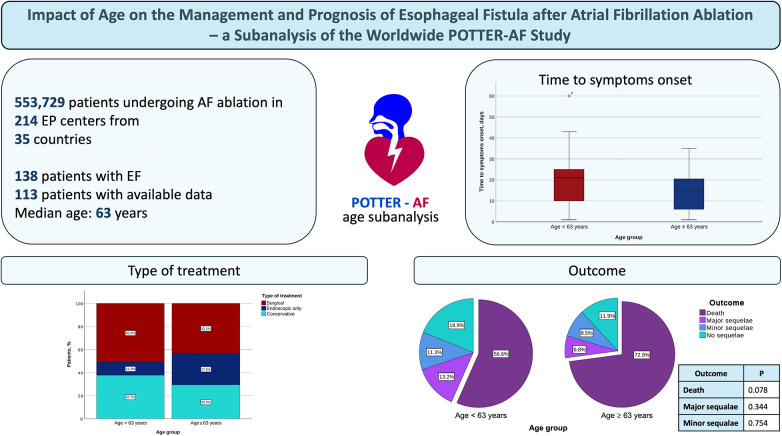
AF, atrial fibrillation; EF, esophageal fistula; EP, electrophysiological.

## Introduction

Atrial fibrillation (AF) is the most common arrhythmia in adults, and its prevalence is expected to double in the next few decades, posing a high burden on the healthcare systems worldwide (1, 2). Catheter ablation is the cornerstone of the rhythm control strategy and is recommended as first-line therapy in patients with paroxysmal AF and second-line therapy in those with persistent AF (2, 3).

Due to solid validation and increased operator experience, thermal ablation remains the most used technology today, with cryoballoon- and radiofrequency-based catheter ablation performed on a wide scale worldwide (4–8). Although the safety profile of these technologies improved over time due to significant technological improvements, thermal-related complications such as pulmonary vein stenosis, phrenic nerve palsy, and esophageal fistulas (EFs) cannot be completely avoided (9–14).

EF is rare but has a high fatality rate (11, 12, 14, 15). The recently published POTTER-AF study was the largest to date to investigate the incidence, management, and prognosis of EF following AF and atrial tachycardia ablation. The study reported an overall EF incidence of 0.025% and an overall fatality of 65.8%, rising up to 89.5% among patients treated conservatively (11). The anatomical proximity between the left atrium and the esophagus is a determining factor in the development of EF, and the lack of fatty tissue between the two structures increases the risk (16). In obese patients, the risk of EF formation is therefore decreased (17). There are several hypotheses on the development of atrioesophageal fistula after catheter ablation including direct thermal effects, ischemia of the esophageal mucosa via occlusion of an esophageal artery, or nerve lesions resulting in motility disorders (16, 17). As an empirical measure, mucosal protection via proton pump inhibitors (PPI) postprocedurally might be efficient in preventing the complication and is widely implemented (3, 18, 19). Esophageal temperature measurement was also suggested to prevent the development of EF; however, the current data are contradictory (14, 20).

One of the most important patient characteristics with a significant impact on ablation success and safety is age. It has been shown that advanced age is associated with a higher risk of atrial arrhythmia recurrence and a higher incidence of major cardiac adverse events (21, 22). However, the impact of age on the management and outcome of EF is unknown.

This subanalysis of the POTTER-AF study aimed to investigate the impact of patients’ age on the management and outcome of EF following catheter ablation for AF.

## Methods

### Study design

The POTTER-AF study is an international, multicenter, anonymized, invitation-based registry, which was conducted at the Department of Rhythmology, University Heart Centre Lübeck under the auspices of the Working Group of Cardiac Electrophysiology of the German Cardiac Society (AGEP, DGK). It was approved by the Ethics Committee of the University of Lübeck, Germany (AZ 21–291), and by the local ethics committees of all participating institutions and was registered at clinicaltrials.gov with the identification number NCT05273645. As this study represents a retrospective analysis of anonymized data, patient consent was not obtained. The study has been conducted in accordance with the ethical standards as laid down in the 1964 Declaration of Helsinki and its later amendments. Experienced electrophysiological centers were invited worldwide (11).

Data were recorded retrospectively and electronically via a standardized online questionnaire using SurveyMonkey. Patients who had an atrioesophageal fistula, esophageal–pericardial fistula, or esophageal perforation after catheter ablation were included in the POTTER-AF study. There were no exclusion criteria.

The present work is an age-based subanalysis of the POTTER-AF study, designed to evaluate the influence of age on the management and outcomes of EF following catheter ablation for AF. In addition, the analysis characterizes age-specific differences in baseline and procedural variables. The study population was divided into two cohorts according to the median age: Group 1 comprised patients younger than the median, and Group 2 included those with a median age or older.

### Statistical analysis

All categorical variables were reported as absolute and relative frequencies and were compared using Fisher's exact test or the *χ*^2^ test, as appropriate. Continuous variables were tested for normal distribution using the Shapiro–Wilk test. They were reported as mean ± standard deviation (SD) in the case of normal distribution, otherwise as median and interquartile range (first quartile, third quartile). Continuous variables were compared using the non-paired Student's *t*-test when normally distributed and the Mann–Whitney *U* test otherwise. A *p*-value of <0.05 was considered statistically significant. All statistical analyses were performed using SPSS version 28.0 (IBM SPSS Statistics)

## Results

### Patient population

**Figure 1:**
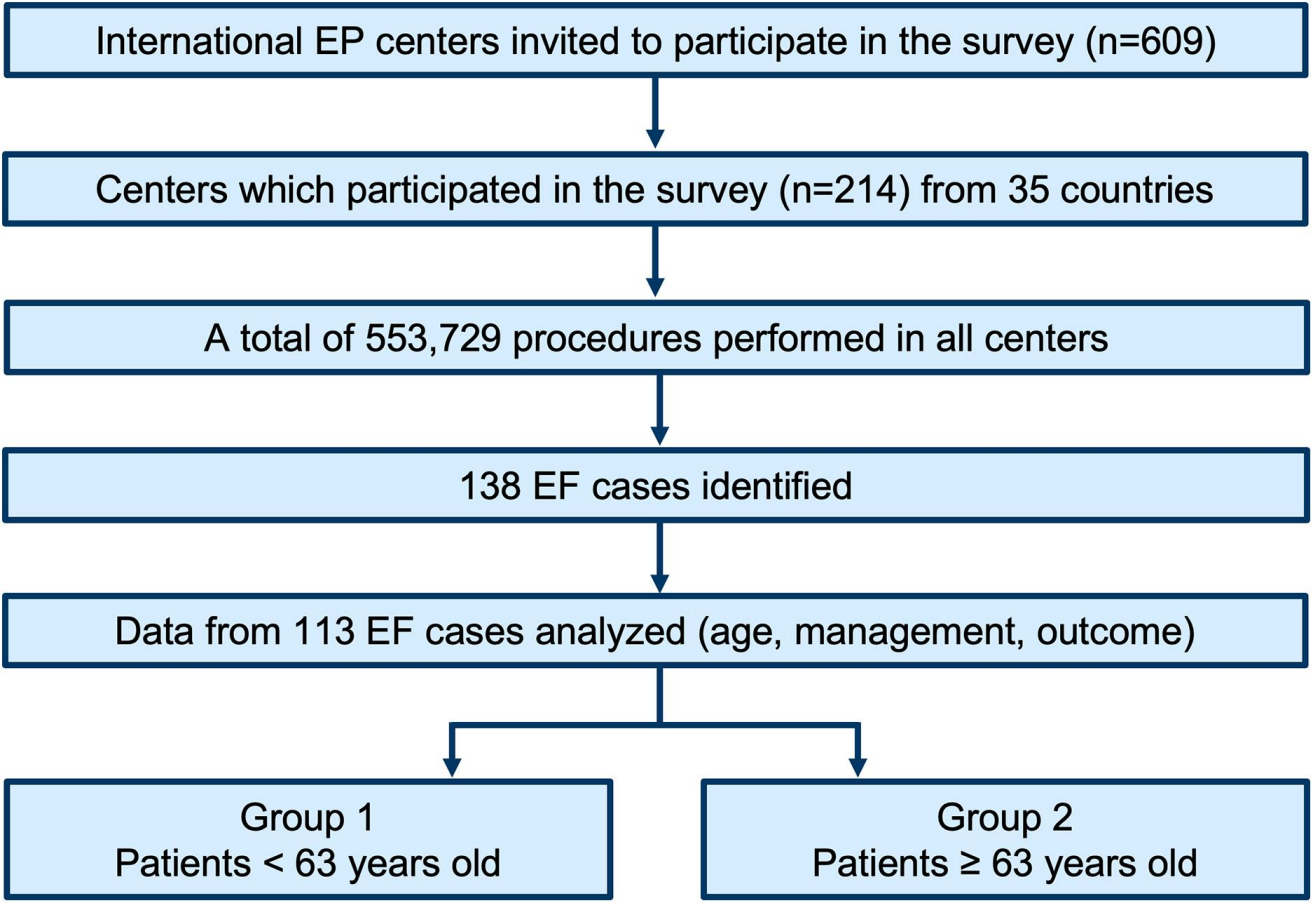
Study flowchart. EF, esophageal fistula; EP, electrophysiological.

A total of 553,729 patients underwent ablation procedures for AF or atrial tachycardia in 214 electrophysiological centers from 35 countries between 1996 and 2022. Of them, 138 (0.025%) patients experienced postprocedural EF, and data regarding the age, management, and prognosis were available in 113 patients. The median age of the population was 63 years. A total of 54 (47.8%) patients experiencing EF were younger than 63 years old (Group 1), and 59 (52.2%) patients were at least 63 years old (Group 2) (Figure 1).

The baseline characteristics of the two groups are depicted in Table 1. Almost half of the patients in each group were females (44.4% vs. 48.3%; *p* = 0.708). No difference was noted between the groups regarding the median body mass index (26.5 vs. 26.3, *p* = 0.813). Regarding the comorbidities, Group 2 was more likely to suffer from coronary artery disease (25.0% vs. 9.8%; *p* = 0.046) and hypertension (72.9% vs. 42.6%; *p* = 0.001). As expected, a CHA_2_DS_2_-VASc score lower than 3 was less frequent in the older population (39.0% vs. 82.7%; *p* < 0.001). There was no statistically significant difference between the two groups regarding the incidence of structural heart disease, congestive heart failure, diabetes, vascular disease, chronic kidney disease, and history of esophageal/gastric disease.

**Table 1:** Baseline characteristics.

Characteristics	Age < 63	Age ≥ 63	*p*-value
Female sex, *n* (%)	24/54 (44.4%)	28/58 (48.3%)	0.708
Body mass index, kg/m^2^	26.5 (24.4, 28.3)	26.3 (22.7, 29.4)	0.813
Structural heart disease, *n* (%)	12/53 (22.6%)	22/59 (37.3%)	0.104
Left ventricular ejection fraction, %	60.0 (50.0, 65.0)	60.0 (52.5, 64.0)	0.837
Coronary artery disease, *n* (%)	5/51 (9.8%)	14/56 (25.0%)	**0** **.** **046**
Congestive heart failure, *n* (%)	7/52 (13.5%)	11/58 (19.0%)	0.607
Hypertension, *n* (%)	23/54 (42.6%)	43/59 (72.9%)	**0** **.** **001**
Diabetes, *n* (%)	8/54 (14.8%)	7/59 (11.9%)	0.783
Vascular disease, *n* (%)	6/41 (14.6%)	8/45 (17.8%)	0.775
Chronic kidney disease, *n* (%)	3/42 (7.1%)	7/46 (15.2%)	0.320
History of esogastric pathology, *n* (%)	5/50 (10%)	3/56 (5.4%)	0.471
CHA_2_DS_2_-VASc score < 3	43/52 (82.7%)	23/59 (39.0%)	**<0** **.** **001**
PPI before ablation, *n* (%)	14/51 (27.5%)	9/55 (16.4%)	0.238
Paroxysmal AF, *n* (%)	23/54 (42.6%)	25/59 (42.4%)	1
Previous AF/atrial tachycardia ablation, *n* (%)	5/54 (9.3%)	5/59 (8.5%)	1

Values are counts, *n* (%), or median and interquartile range (Q1, Q3) as appropriate. PPI, proton pump inhibitors; AF, atrial fibrillation.

Bold indicates statistically significant values.

### Symptom onset and diagnosis

**Figure 2:**
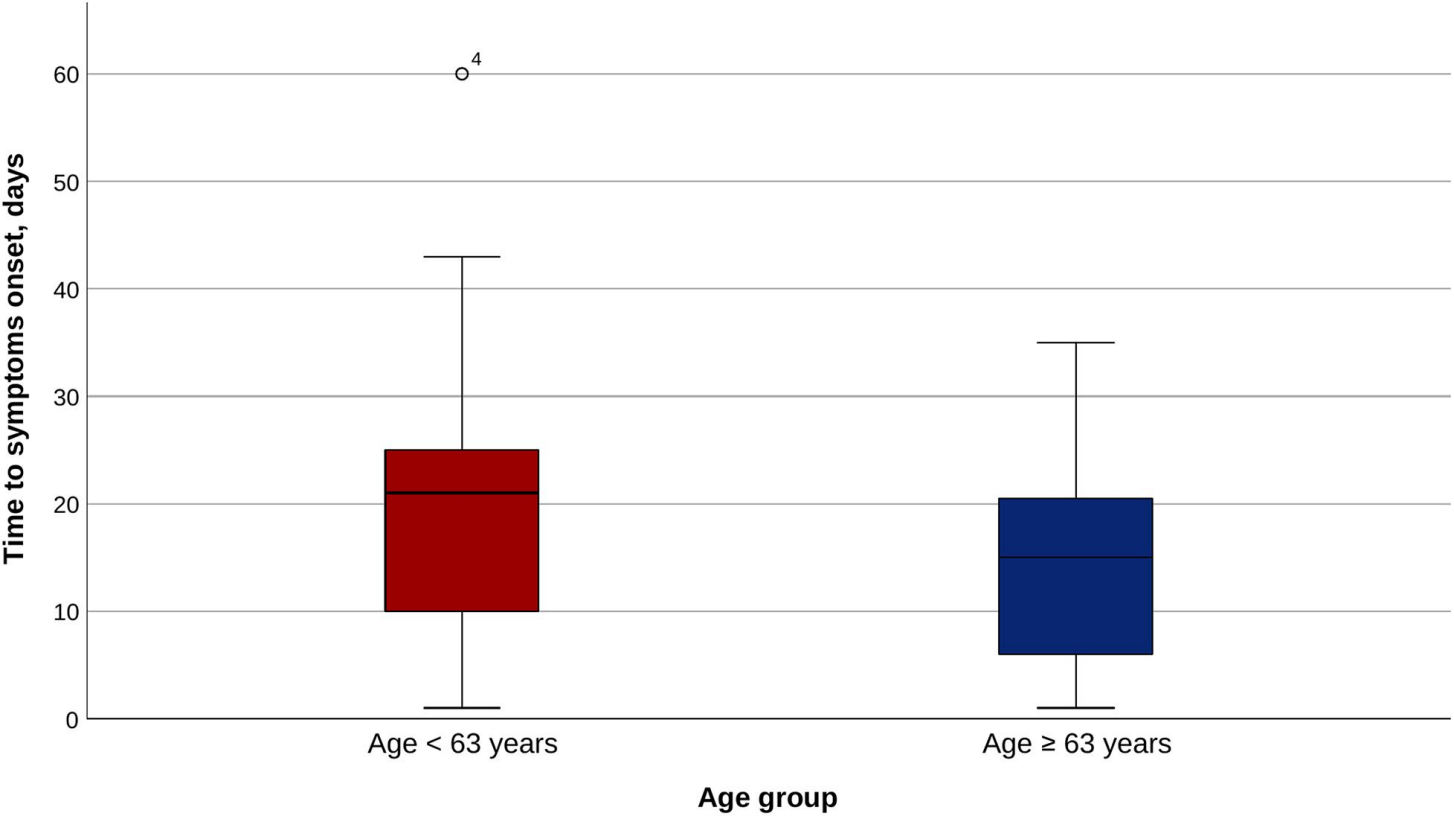
Time to symptom onset in the two age groups.

The median time to symptom onset was 21.0 (10.0, 25.3) days in Group 1 and 15.0 (6.0, 21.0) days in Group 2 (*p* = 0.031), while the median time to EF diagnosis was 23.0 (14.5, 32.0) days and 19.0 (14.3, 29.0), respectively (*p* = 0.240; Figure 2). There was no significant difference between the groups regarding the initial symptoms, as well as regarding the further complications developed (Table 2).

**Table 2:** Initial symptoms and further complications.

Characteristics	Age < 63	Age ≥ 63	*p*-value
Initial symptoms			
Duration until initial symptoms, days	21.0 (10.0, 25.3)	15.0 (6.0, 21.0)	**0** **.** **031**
Duration until EF diagnosis, days	23.0 (14.5, 32.0)	19.0 (14.3, 29.0)	0.240
Duration from initial symptoms until EF diagnosis, days	2.0 (1.0, 6.0)	4.0 (0.5, 10.5)	0.411
Duration from procedure until hospital admission, days	21.0 (12.0, 26.8)	18.5 (12.3, 27.0)	0.763
Fever, *n* (%)	33/54 (61.1%)	34/59 (57.6%)	0.848
Chest pain/odynophagia, *n* (%)	29/54 (53.7%)	31/59 (52.5%)	1
Neurological signs, *n* (%)	26/54 (48.1%)	25/59 (42.4%)	0.574
Other symptoms, *n* (%)	30/54 (55.6%)	44/59 (74.6%)	0.047
Further complications			
Stroke, *n* (%)	10/48 (20.8%)	14/54 (25.9%)	0.642
Septic shock, *n* (%)	26/48 (54.2%)	32/54 (59.3%)	0.690
Coma, *n* (%)	22/48 (45.8%)	25/54 (46.3%)	1
Cardiac arrest, *n* (%)	9/48 (18.8%)	11/54 (20.4%)	1
Tamponade, *n* (%)	6/48 (12.5%)	4/54 (7.4%)	0.510
Gastrointestinal bleeding, *n* (%)	10/48 (20.8%)	8/54 (14.8%)	0.448
Other complications, *n* (%)	14/48 (29.2%)	16/54 (29.6%)	1

Values are counts, *n* (%), or median and interquartile range (Q1, Q3) as appropriate. EF, esophageal fistula.

Bold indicates statistically significant values.

When analyzing the diagnostic methods, Group 2 was less likely to undergo a brain computed tomography (CT) or brain magnetic resonance imaging (MRI) (25.9% vs. 45.3%; *p* = 0.046). There was no statistically significant difference between the use of chest CT, endoscopy, or echocardiography (Table 3).

**Table 3:** Diagnosis methods and type of esophageal fistula.

Characteristics	Age < 63	Age ≥ 63	*p*-value
Diagnostic method			
Chest CT, *n* (%)	43/53 (81.1%)	46/58 (79.3%)	1
Endoscopy, *n* (%)	11/53 (20.8%)	13/58 (22.4%)	1
Echocardiography, *n* (%)	12/53 (22.6%)	16/58 (27.6%)	0.663
Brain CT or brain MRI, *n* (%)	24/53 (45.3%)	15/58 (25.9%)	**0**.**046**
Others, *n* (%)	12/53 (22.6%)	7/58 (12.1%)	0.207
Type of esophageal fistula			
Atrioesophageal fistula	52/54 (96.3%)	56/59 (94.9%)	1
Esophageal perforation	0/54 (0.0%)	1/59 (1.7%)	1
Esophageal–pericardial fistula	2/54 (3.7%)	2/59 (3.4%)	1

Values are counts, *n* (%). CT, computed tomography; MRI, magnetic resonance imaging.

Bold indicates statistically significant values.

No difference was noted between the groups regarding the proportions of atrioesophageal fistula, esophageal perforation, and esophageal–pericardial fistula (Table 3).

### Procedural characteristics

The two populations were similar in terms of sedation type, energy source, and ablation techniques used, as well as in terms of esophageal temperature probe utilization (Table 4). General anesthesia was used in 44.4% of patients in Group 1 vs. 49.2% of patients in Group 2 (*p* = 0.707). Most of the patients underwent a radiofrequency-based catheter ablation (94.4% in Group 1 vs. 98.3% in Group 2; *p* = 0.347). An esophageal temperature probe was used in 24.1% of patients in Group 1 and in 25.4% of patients in Group 2 (*p* = 1).

**Table 4:** Procedural characteristics.

Characteristics	Age < 63	Age ≥ 63	*p*-value
Conscious sedation, *n* (%)	11/54 (20.4%)	6/59 (10.2%)	0.188
Deep analgosedation, *n* (%)	15/54 (27.8%)	19/59 (32.2%)	0.683
General anesthesia, *n* (%)	24/54 (44.4%)	29/59 (49.2%)	0.707
Use of thermal probe, *n* (%)	13/54 (24.1%)	15/59 (25.4%)	1
Procedure duration, minutes	141.5 (105.0, 183.0)	148.5 (112.5, 180.0)	0.895
Radiofrequency, *n* (%)	51/54 (94.4%)	58/59 (98.3%)	0.347
Cryoballoon, *n* (%)	2/54 (3.7%)	1/59 (1.7%)	0.605
Laser balloon, *n* (%)	1/54 (1.9%)	0/59 (0.0%)	0.478
RF duration, minutes	38.5 (25.3, 59.1)	38.0 (21.0, 48.0)	0.346
PVI circumferential, *n* (%)	48/49 (98.0%)	50/55 (90.9%)	0.210
PVI segmental ostial, *n* (%)	1/49 (2.0%)	0/55 (0.0%)	0.471
PVI anatomical, *n* (%)	1/49 (2.0%)	5/55 (9.1%)	0.210
PPI postprocedural, *n* (%)	39/50 (78.0%)	42/59 (71.2%)	0.511

Values are counts, *n* (%), or median and interquartile range (Q1, Q3) as appropriate. RF, radiofrequency; PVI, pulmonary vein isolation; PPI, proton pump inhibitors.

### Management and outcome

**Figure 3:**
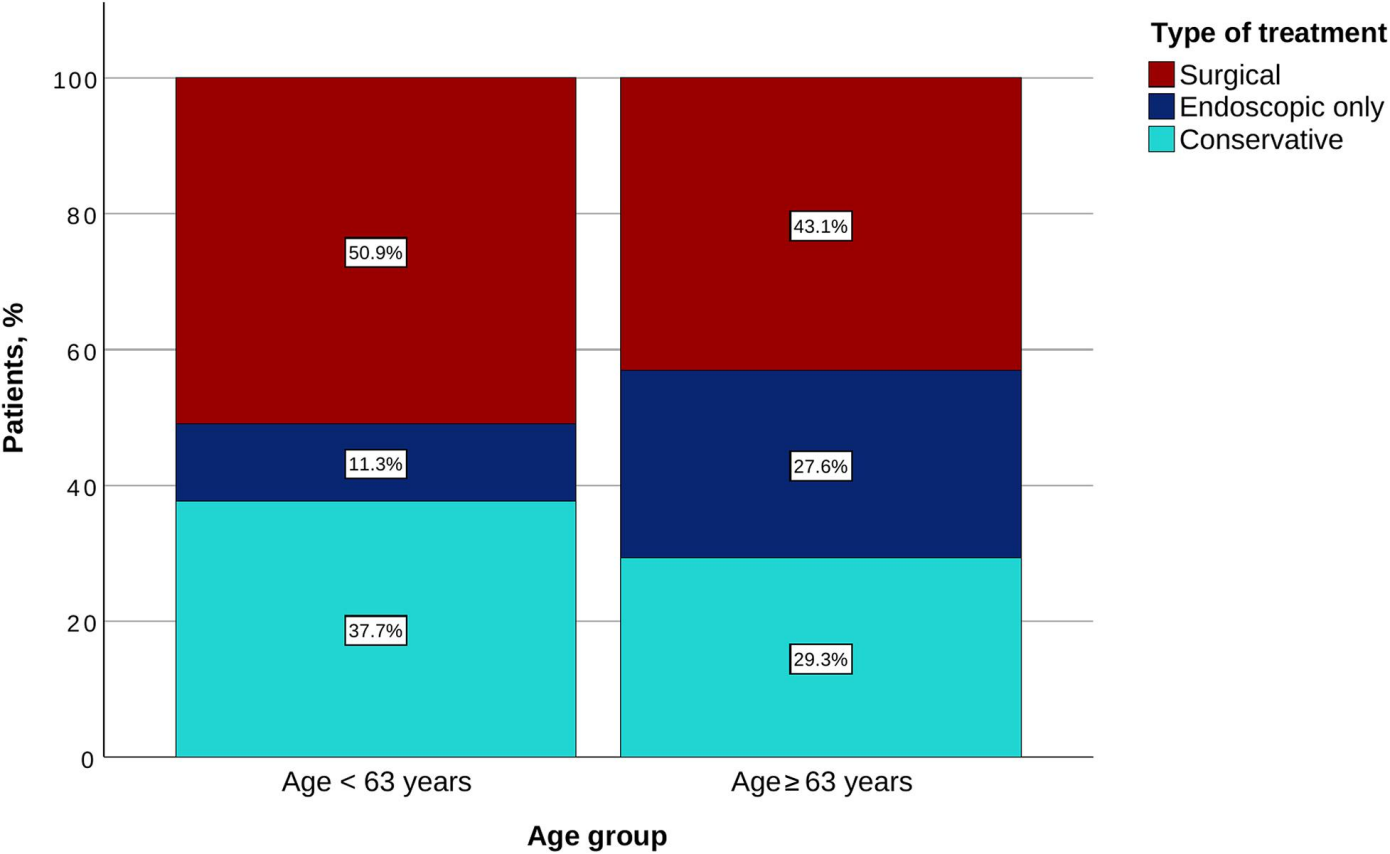
Type of treatment for each age group.

**Figure 4:**
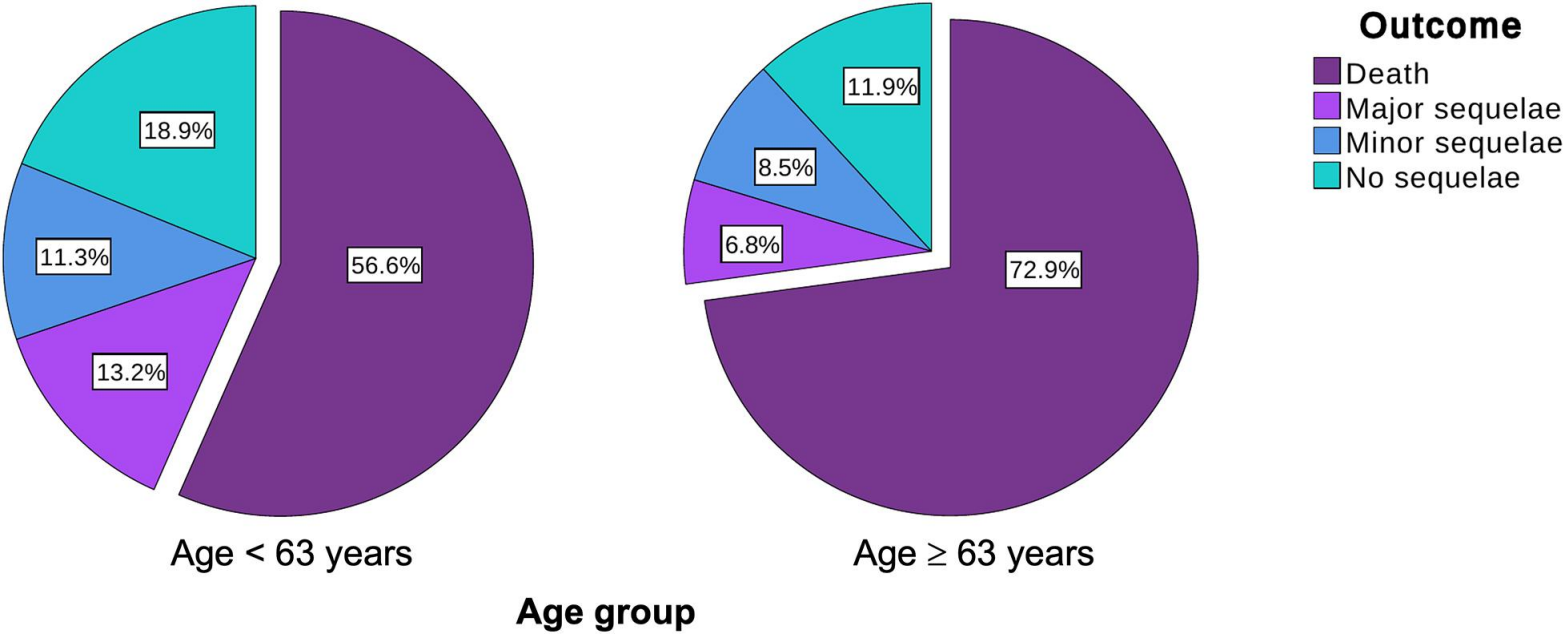
Outcome for each age group.

The older population was more likely to undergo a direct endoscopic treatment without surgery (27.6% vs. 11.3%; *p* = 0.035), while there was no difference regarding the rate of surgical treatment. The conservative treatment was used in similar proportions (37.7% in Group 1 vs. 29.3% in Group 2; *p* = 0.421) (Table 5 and Figure 3). A clear trend toward a higher fatality was noted in the older population (72.9% vs. 56.6%; *p* = 0.078). The incidence of major and minor sequelae was similar for both groups (Table 5 and Figure 4).

**Table 5:** Management.

Characteristics	Age < 63	Age ≥ 63	*p*-value
Esophageal surgery, *n* (%)	27/53 (50.9%)	25/58 (43.1%)	0.450
Esophageal surgery without endoscopic treatment, *n* (%)	22/53 (41.5%)	23/58 (39.7%)	0.849
Endoscopic treatment, *n* (%)	11/53 (20.8%)	18/58 (31.0%)	0.281
Endoscopic treatment without surgery, *n* (%)	6/53 (11.3%)	16/58 (27.6%)	0.035
Conservative treatment, *n* (%)	20/53 (37.7%)	17/58 (29.3%)	0.421

Values are counts, *n* (%). Under “endoscopic treatment” are reported all patients who underwent any endoscopic intervention, irrespective of whether they also received surgical treatment. Under “esophageal surgery” are reported all patients who underwent any surgical intervention, regardless of concurrent endoscopic treatment. The subcategories listed with bullet points indicate the number of patients in each group who received exclusively endoscopic or exclusively surgical treatment.

The fatality of EF was significantly higher in patients managed conservatively compared with that of patients treated invasively (endoscopically and/or surgically) in both Group 1 (80.0% vs. 40.6%, *p* = 0.009) and Group 2 (100.0% vs. 61.0%, *p* = 0.003).

## Discussion

To the best of our knowledge, this is the first study to date to evaluate the impact of age on EF management and prognosis following catheter ablation for AF or AT. The main findings of the study are as follows:

1. The time to symptom onset was significantly shorter in the older population.
2. Brain CT or MRI was less commonly used among older patients.
3. Direct endoscopic treatment was used more often in the older population.
4. Conservative therapy was used in similar proportions of patients.
5. The older patients showed a trend toward a higher fatality.

Age is the main risk factor for developing AF and for its progression from paroxysmal to persistent type (1). In some studies, age is shown to predict the postprocedural complication rates after AF ablation; however, the current data are contradictory (21–28).

Regarding EF following catheter ablation for AF, it has been shown that the treatment, either endoscopic or surgical, can improve the outcome in these patients (11, 14, 29, 30). For this reason, the early diagnosis and initiation of therapy are crucial to reduce mortality. An important limitation in this case is the relatively late onset of symptoms following AF ablation, which can lead to a delayed diagnosis (11, 14, 29, 30). As demonstrated in the POTTER-AF study, the patients with an early EF detection received an early treatment via endoscopy or esophageal surgery and showed a lower fatality as compared with those with late detection, who more often received a conservative treatment (11). The present study demonstrated a shorter time to symptom onset in the elderly population, which might be at least partially explained by the frailer status of these patients. However, this difference did not translate into a lower fatality in this group, probably due to the impact of age itself on the prognosis.

When a clinical suspicion for EF occurs, the most frequently used modality for diagnosis is chest CT (11, 22, 29, 30). Transthoracic echocardiography is not sensitive enough, and transesophageal echocardiography should be avoided, as it may worsen the situation (30, 31). Esophagogastroscopy or nasogastric tube insertion might also be detrimental, as it can open the tissue flap and increase the blood flow through the fistula and aggravate systemic air embolism (32). In the present study, the most common diagnostic method was the chest CT, with similar rates in both groups. However, in the elderly population, the rate of brain CT and brain MRI was lower compared with the younger population, although the incidence of neurological symptoms was similar between the groups.

In the present study, direct endoscopic treatment without surgery was significantly more often used in the elderly group, while the surgical treatment was slightly more common in the younger population, without reaching the statistical significance level. This observation might however be biased by the clinical status of the patients, which is expected to be more deteriorated in the older group, leading to a lower rate of surgical interventions. As previously discussed, the use of a conservative approach was associated with a higher fatality as compared with endoscopic or surgical approaches (11, 12, 29, 30). In this study, the use of a conservative approach was slightly more common in the younger population, without reaching statistical significance. Given the retrospective nature of this study, the criteria guiding treatment decisions cannot be derived from our dataset. Nevertheless, several hypotheses may help explain this counterintuitive observation. One possibility is that younger patients appeared clinically more stable at presentation, prompting clinicians to opt for a conservative strategy. However, considering the high lethality of this condition, such an approach may be misleading, as conservatively managed patients generally exhibit poorer outcomes. Another contributing factor could be a longer time from symptom onset to diagnosis observed in younger individuals, potentially influencing clinical decision-making. A further explanation may relate to the perceived greater physiological reserve in younger patients, leading to the expectation that they might recover without aggressive intervention.

A trend toward higher fatality (i.e., the proportion of patients who died from EF among those who developed it) was observed in the older population. This finding should be taken into account when discussing the risks and benefits of catheter ablation for AF in this age group.

Regardless of age, it is important to emphasize that conservative management is associated with a worse prognosis, and an interventional or surgical approach should be pursued whenever feasible (11, 14).

### Limitations

This is a subanalysis of a retrospective, invitation-based, international registry and comes with several specific limitations. First, the incidence of EF in the two age-based groups could not be determined due to the study design. Of note, the prevalence of mediastinal changes diagnosed by endosonography was not age dependent, so we cannot exclude that the observed age differences represent an accidental observation (33). Second, only data from patients exhibiting EF were collected, so the assessment of predictors of EF occurrence was not possible. Third, the decision regarding the management, as well as the outcome, could have been biased due to comorbidities, critical illness, and operability of the patients. Data on preoperative physical health status, such as the American Society of Anesthesiologists (ASA) score, which could have provided valuable insights into the decision-making process, were not available for this analysis. Fourth, due to the retrospective character of the registry, not all data were available for the whole population. Fifth, given the method of data acquisition via an invited survey, underreporting of these complications and the loss of detailed individual patient information cannot be excluded.

## Conclusions

This is the first study to date to report on the impact of age on the management and outcome of EF following catheter ablation for AF. Older patients had initial symptoms earlier, and brain CT or brain MRI was used less commonly as a diagnostic modality in this population. Older patients who developed EF after catheter ablation were more frequently treated with direct endoscopic interventions and showed a trend toward higher fatality.

## Data Availability

The datasets presented in this article are not readily available because data supporting the POTTER-AF study are curated at the Clinical Trial Unit of the Department of Rhythmology, University Hospital Schleswig-Holstein, Germany. These data are not shared openly but are available on reasonable request from the corresponding authors. Requests to access the datasets should be directed to RT, tilz6@hotmail.com.
